# Development and Validation of a Gene Expression Score That Predicts Response to Fulvestrant in Breast Cancer Patients

**DOI:** 10.1371/journal.pone.0087415

**Published:** 2014-02-05

**Authors:** Steen Knudsen, Thomas Jensen, Anker Hansen, Wiktor Mazin, Justin Lindemann, Irene Kuter, Naomi Laing, Elizabeth Anderson

**Affiliations:** 1 Medical Prognosis Institute, Hørsholm, Denmark; 2 Astrazeneca UK Limited, Oncology iMED, Alderley Park, Cheshire, United Kingdom; 3 Astrazeneca R&D Boston, Waltham, United States of America; 4 Massachusetts General Hospital, Massachusetts, Boston, United States of America; 5 Now at Boehringer-Ingelheim RCV GmbH & Co KG, Vienna, Austria; 6 Now at the Department of Clinical Epidemiology at Aarhus University Hospital, Aarhus C, Denmark; University of Wisconsin – Madison, United States of America

## Abstract

Fulvestrant is a selective estrogen receptor antagonist. Based on the measured growth inhibition of 60 human cancer cell lines (NCI60) in the presence of fulvestrant, as well as the baseline gene expression of the 60 cell lines, a gene expression score that predicts response to fulvestrant was developed. The score is based on 414 genes, 103 of which show increased expression in sensitive cell lines, while 311 show increased expression in the non-responding cell lines. The sensitivity genes primarily sense signaling through estrogen receptor alpha, whereas the resistance genes modulate the PI3K signaling pathway. The latter genes suggest that resistance to fulvestrant can be overcome by drugs targeting the PI3K pathway. The level of this gene expression score and its correlation with fulvestrant response was measured in a panel of 20 breast cancer cell lines. The predicted sensitivity matched the measured sensitivity well (CC = −0.63, P = 0.003). The predictor was applied to tumor biopies obtained from a Phase II clinical trial. The sensitivity of each patient to treatment with fulvestrant was predicted based on the RNA profile of the biopsy taken before neoadjuvant treatment and without knowledge of the subsequent response. The prediction was then compared to clinical response to show that the responders had a significantly higher sensitivity prediction than the non-responders (P = 0.01). When clinical covariates, tumor grade and estrogen receptor H-score, were included in the prediction, the difference in predicted senstivity between responders and non-responders improved (P = 0.003). Using a pre-defined cutoff to separate patients into predicted sensitive and predicted resistant yielded a positive predictive value of 88% and a negative predictive value of 100% when compared to clinical data. We conclude that pre-screening patients with the new gene expression predictor has the potential to identify those postmenopausal women with locally advanced, estrogen-receptor-positive breast cancer most likely to respond to fulvestrant.

## Introduction

Fulvestrant is a highly selective oestrogen receptor (ER) antagonist with no known agonist effects that is approved for the treatment of ER-positive metastatic breast cancer in post-menopausal women with disease progression following anti-oestrogen therapy. A number of pre-clinical investigations followed by pre-surgical studies in ER-positive primary breast cancer patients have shown that treatment with fulvestrant acts primarily by down-regulating ER protein in a dose-dependent manner. This is accompanied by depletion of the ER-regulated protein, progesterone receptor (PgR), and a reduction in proliferative activity as indicated by the Ki67 labelling index. This mechanism of action differs from that of tamoxifen which is a selective oestrogen receptor modulator and also from that of aromatase inhibitors such as anastrozole, letrozole and exemestane which inhibit synthesis of oestradiol, the endogenous ER ligand. Fulvestrant's distinct mechanism of action is thought to contribute to its efficacy in patients who have progressed following treatment with the other endocrine agents [1]. Furthermore, in the first line setting, there is some evidence to suggest that fulvestrant may be more effective than the aromatase inhibitors which are currently standard of care. The data from the FIRST trial, a randomised, phase II study comparing fulvestrant to anastrazole in the first line treatment of postmenopausal women with hormone receptor positive advanced breast cancer, showed that the time to progression was 23.4 months for the Fulvestrant 500 mg arm compared with 13.1 months for anastrozole (HR = 0.66, 95% CI 0.47 – 0.92; p = 0.01 [2]). Nevertheless and as is the case for other endocrine agents, it is clear that not all patients with ER-positive tumours respond to treatment with fulvestrant. There is apparently a subset of patients who derive substantial benefit and others who will not respond at all. There is a clear need for a means by which these subgroups of patients with ER-positive breast cancer can be identified and directed toward appropriate therapy.

Measurement of single biomarkers such as the ER or HER-2, a member of the epidermal growth factor receptor (EGFR) family, are used in standard care to identify patients more likely to respond to anti-oestrogens such as tamoxifen or HER-2 targeted therapies such as trastuzumab (Herceptin™). Since the cure rates for these targeted therapies in biomarker positive patients are less than optimal, biomarkers that can predict the potential to respond to the available therapies for breast cancer may aid in improving treatment decisions. Several methods capable of simultaneously measuring multiple biomarkers in small samples of tumour tissue are now being developed and some have been introduced into the management of breast cancer. One such assay is the Oncotype Dx test which has been demonstrated to have prognostic value in that it can identify primary breast cancer patients at increased risk of early recurrence [3]. However, diagnostic assays that can more accurately predict response to specific treatments have yet to make an impact on the routine clinical management of breast cancer.

The NEWEST (Neoadjuvant Endocrine Therapy for Women with Estrogen-Sensitive Tumours) trial compared the clinical and biological activity of fulvestrant 500 mg vs 250 mg in the neoadjuvant setting. In this multi-centre phase II study, post-menopausal women with operable, locally advanced (T2, 3, 4b; N0-3; M0) ER-positive breast tumours were randomised to receive neoadjuvant treatment with either dose of fulvestrant for 16 weeks before surgery [4]. Tumour core biopsies were obtained at baseline, 4 weeks and at surgery for assessment of changes in biomarker expression. Tumour volumes were measured by 3–D ultrasound at the same timepoints. In this trial, the percentage of patients who showed a reduction in tumour volume or stabilisation of disease (using RECIST criteria) after treatment with fulvestrant 500 mg was 36% (26 out of 69 patients). Therefore, within a population of endocrine–therapy naive patients whose tumours were confirmed as being ER–positive at the time of study entry, there is a subgroup who gained particular clinical benefit from fulvestrant treatment. These clinical response data together with the availability of biological response information and frozen tumour tissue from participants makes the NEWEST trial an attractive setting in which to investigate the potential of new markers of response to fulvestrant.

In the present study, we describe the development of a pre-treatment gene expression score that predicts response to fulvestrant 500 mg. This is based on the identification of a number of genes across the NCI60 cancer cell line panel, whose baseline expression correlate with sensitivity to fulvestrant treatment in vitro. The expression of the resulting gene expression score was further validated in a blinded fashion in an extended panel of breast cancer cell lines. Finally, the gene expression score was determined in the tumour samples available from the NEWEST study and correlated with reduction in tumour size. A substantial proportion of patients whose tumours were predicted to be sensitive to fulvestrant and who received the 500 mg dose of fulvestrant had partial responses (7 out of 8 by RECIST criteria). There was further evidence of the dose dependency of fulvestrant efficacy in the fact that patients predicted to be sensitive to fulvestrant but who received the lower dose of fulvestrant generally only showed disease stabilisation (9 out of 12). Most patients whose tumours expressed lower than the median level of the sensitivity score failed to respond to fulvestrant irrespective of dose received (13 out of 19 patients).

## Materials and Methods

### Ethics statement

The study was conducted in accordance with the Declaration of Helsinki and with local ethics committee approval at each participating center. The primary IRB was Dana Farber/Harvard Institutional Review Board. Written consent was obtained from each patient prior to enrollment in the study; it was explicitly stated in the consent that the information would be stored and used for research.

The consent form explained that “medical information created by this study may become part of your medical record.” The patients were informed that their protected health information may be shared with the sponsor of the study, AstraZeneca.

### Patients

NEWEST (Neoadjuvant Endocrine Therapy for Women with Estrogen-Sensitive Tumors; 9238IL/0065) was a randomized, open-label, multicenter, Phase II study involving postmenopausal women with newly diagnosed, ER-positive, locally advanced breast cancer who had received no prior breast cancer treatment (NCT0093002). Eligible patients were randomized 1∶1 to receive either fulvestrant 500 mg or 250 mg for 16 weeks preceding surgery. Fulvestrant 500 mg was given as two 5 ml intramuscular injections on days 0, 14, 28 and every 28 days thereafter for 16 weeks. Fulvestrant 250 mg was given as one 5 ml injection on days 0, 28 and every 28 days thereafter for 16 weeks [4].

A total of 211 patients were enrolled in NEWEST. Of these, 173 completed the study and 121 met all protocol criteria. Fresh frozen pre-treatment samples with sufficient RNA (100 ng) was available from 44 patients. Two samples were discarded due to array quality (see below), leaving 42 patients. Of these 42 patients, 22 were in the 500 mg treatment group and 20 were in the 250 mg treatment group.

During the 16-week treatment phase, patients underwent clinical breast examination every 4 weeks. Tumor volume was measured by 3D ultrasound at baseline, week 4, and after 16 weeks of treatment before definitive surgery. Tumor response was defined as complete response (CR, disappearance of all lesions), partial response (PR, at least 65% reduction in tumor volume by 3D ultrasound), disease progression (PD, at least 73% increase in tumor volume), or stable disease (SD, neither partial response nor disease progression). The reduction for partial response was in relation to baseline but for progressive disease was in relation to the smallest tumour volume at any preceeding assessment. Objective responders were those patients with a complete response or partial response.

Of the 42 patients available for array analysis, 15 patients had partial responses, 22 patients had stable disease, 2 patients had progressive disease and 3 patients were not evaluable.

ER H score was derived by immunohistochemical staining using the 1D5 anti-ER antibody (Dako) followed by microscopic assessment of the percentage of tumor cells in each of five staining categories (negative, very weak, weak, moderate and strong) to give an H score ranging from 0 to 300.

Trial 223 [5] was a placebo-controlled trial of neoadjuvant anastrozole alone or with gefitinib in early breast cancer. We used patients from arm B and C: anastrozole 1 mg/d for the duration of the 16 week period plus placebo 1 tablet/d orally for 2 weeks. Patients in arm B were followed by gefitinib 250 mg/d orally for 14 weeks whereas patients in arm C continued with placebo for 14 weeks. We received pre-treatment biopsy material from 21 patients in arms B and C with Ki67 measurements after 2 weeks. We received pre-treatment biopsy material from 16 patients in arm C with outcome information (RECIST).

### Microarray analysis

44 RNA samples from NEWEST and 21 samples from 223 were run on Affymetrix HG-U133_Plus_2 arrays after amplification using Ambion MessageAmp Premier following the manufacturer's instructions. The quality criteria used for accepting RNA was a minimum of 100 ng of total RNA which amplified into a minimum of 5 µg aRNA. 42 arrays from NEWEST and 21 samples from 223 passed quality criteria and a visual inspection of array images for spatial artefacts. All array data have been deposited at GEO under accession numbers GSE48905 and GSE48906.

### Predictor development based on in vitro assays

The in vitro based method of developing a predictor of drug response has been described before, both for pre-clinical use [6] and for clinical use [7]. The growth inhibition values (GI_50_) of 60 cell lines subjected to fulvestrant treatment were downloaded from the DTP web site (http://dtp.nci.nih.gov). Included in the 60 cell lines are 6 derived from breast cancer of which 2 are ERα positive and 4 are ERα negative. In this NCI drug screen, cells are grown in RPMI1640 supplemented with 5% fetal bovine serum, a source of estrogen, and with fulvestrant added in concentrations of 10^−8^ M to 10^−4^ M in order to calculate GI_50_. Baseline gene expression measurements on the Affymetrix GeneChip platform of the same cell lines, in the absence of fulvestrant, were obtained from [8]. Gene expression measurements were logit normalized, that is, for each array the transformation logit = log[(x−background)/(saturation−x)] was carried out followed by a Z-transformation to mean zero and SD 1, and correlated to growth inhibition (−log(GI_50_)). Genes with a Pearson correlation above 0.25 or below −0.25 were considered biomarkers of sensitivity and resistance to later treatment with fulvestrant, respectively, and retained as a response profile for fulvestrant. The entire process was repeated for the other drugs tamoxifen, anastrozole, raloxifene and toremifene to yield a response profile for each drug.

To reduce the number of false positive markers passing the Pearson correlation cutoff, we applied a biological relevance filter that only retained markers already known to interact, similar to the approach described by [9].

The response profile for fulvestrant consisted of 103 positively correlated probesets and 311 negatively correlated probesets and was locked before unblinding of the clinical data.

### Pathway analysis

The probesets that correlated to fulvestrant sensitivity were converted to gene symbols and submitted to g:Profiler [10] for association to pathways and gene ontologies (GO). In addition, the probesets that had a correlation to fulvestrant sensitivity above 0.4 or below −0.4 were manually searched in PubMed and NCBI Gene (http://www.ncbi.nlm.nih.gov/) for reported associations to pathways.

### Prediction of fulvestrant sensitivity in clinical samples

After robust multi-array average (RMA [11]) normalization of array data from the clinical samples, the expression of each gene in the response profile was used to predict sensitivity: *Prediction score = mean(positively correlated genes) -mean(negatively correlated genes).* That means that each gene in the profile is given equal weight. Next, the prediction score was normalized to a scale from 0 to 100 by a linear transformation of the prediction score of all patient samples.

### Prediction of intrinsic subtypes and risk of relapse

The intrinsic subtypes luminal A, luminal B, Her2, normal and basal were predicted using the PAM50 centroids calculated by [12] using the genefu package from www.bioconductor.org. The Risk of Relapse (ROR-S) score was calculated as described by [12]. The Oncotype DX recurrence score was calculated using the gene list and risk function provided in genefu.

### Statistical analysis

The statistical analysis of predicted and observed sensitivity to fulvestrant in patients was performed according to a Statistical Analysis Plan with a pre-specified cutoff equal to the population median and success criteria that were agreed upon before unblinding of the clinical data. The primary analysis was one-sided Wilcoxon test for difference in prediction score between objective responders (PR) and non-responders (SD+PD) and a Pearson correlation between prediction score and reduction in Ki67 at 4 weeks compared to baseline. Clinical covariates specified in the statistical analysis plan were combined with the prediction score without bias by giving equal weight to the prediction score and each of the clinical covariates available: Combination score = Prediction score +30 * tumor grade + ER Hscore/3. Cutoff between predicted sensitive and predicted resistant to fulvestrant was pre-defined as the population median of the prediction score. The population median is taken over all samples, from both dose groups. Areas under the curve (AUC) in a receiver-operating characteristic (ROC) were calculated with the pROC package from www.r-project.org with confidence intervals calculated using the Delong method [13].

## Results

### Response predictor genes and their biological interpretation

A total of 103 probesets correlated to fulvestrant sensitivity in the NCI60 cell lines. That means that they are higher expressed in cell lines that are sensitive to fulvestrant than in cell lines that are resistant. The 103 probesets mapped to 83 unique genes shown in Table 1. In this table they are grouped according to functional associations. Some of these sensitivity genes (higher expression in fulvestrant sensitive cell lines) sense the signaling through estrogen receptor alpha, either because they have Estrogen Responsive Elements (ERE) in their promoters, and/or through microRNA regulation, or though direct interaction with the estrogen-ER complex [14]–[18]. Other genes are associated to broader gene ontology definitions of cell activation and response to stimulus. A number of genes have associations to immune system and cell adhesion (not shown). Note that groups may be overlapping.

**Table 1:** Functional grouping of fulvestrant sensitivity genes.

Function	Genes
Estrogen signaling	GATA3, TFF1, BCL2, NHERF1, PDCD4
GO cell activation	GATA3, CD8B1, BCL2, CD37, SPI1, DGKZ, SELPLG, ICOS, FLT3LG, CD28, CD48, ITGAM
GO response to stimulus	GATA3, TFF1, CD8B1, BCL2, FBP1, ITGB7, PDCD4, CD37, ORM1, DGKZ, SELPLG, ICOS, FLT3LG, HCLS1, PTGER3, CD28, BIN2, TOB1, ATP2A3, MFNG, TNFRSF25, IGLL1, SIPA1L3, NUP210, CISH, IFI30, CTSS, CD48, IGFBP5, RNASE6, CLIC3, DOK2, GLUL, ICAM3, ITGAM, IGJ, ICAM2, DMBT1, HSPA6, PACAP
Other	CBFA2T3, SPDEF, HBA1, TBC1D30, VNN2, HIST1H3H, HBA2, TARP, KIAA0182, PTP4A3, JUP, PSCD4, HEM1, GIMAP4, HAB1, SIRPB2, PNAS-4, TRDD3, CIZ1, CLDN3, HIST1H2BG, SLC39A6, FMO5, ASS, LRMP, MCCC2, MAGEA9, MYLIP, CABC1, MDS028, LOC81558, GALNT6, FOXO1A, DHCR7, SSBP2, BG1, ZNF165, C1orf38, TETRAN, TRA@, PSTPIP2, PIM2, ZNF394, ABCA7

**Figure 1:**
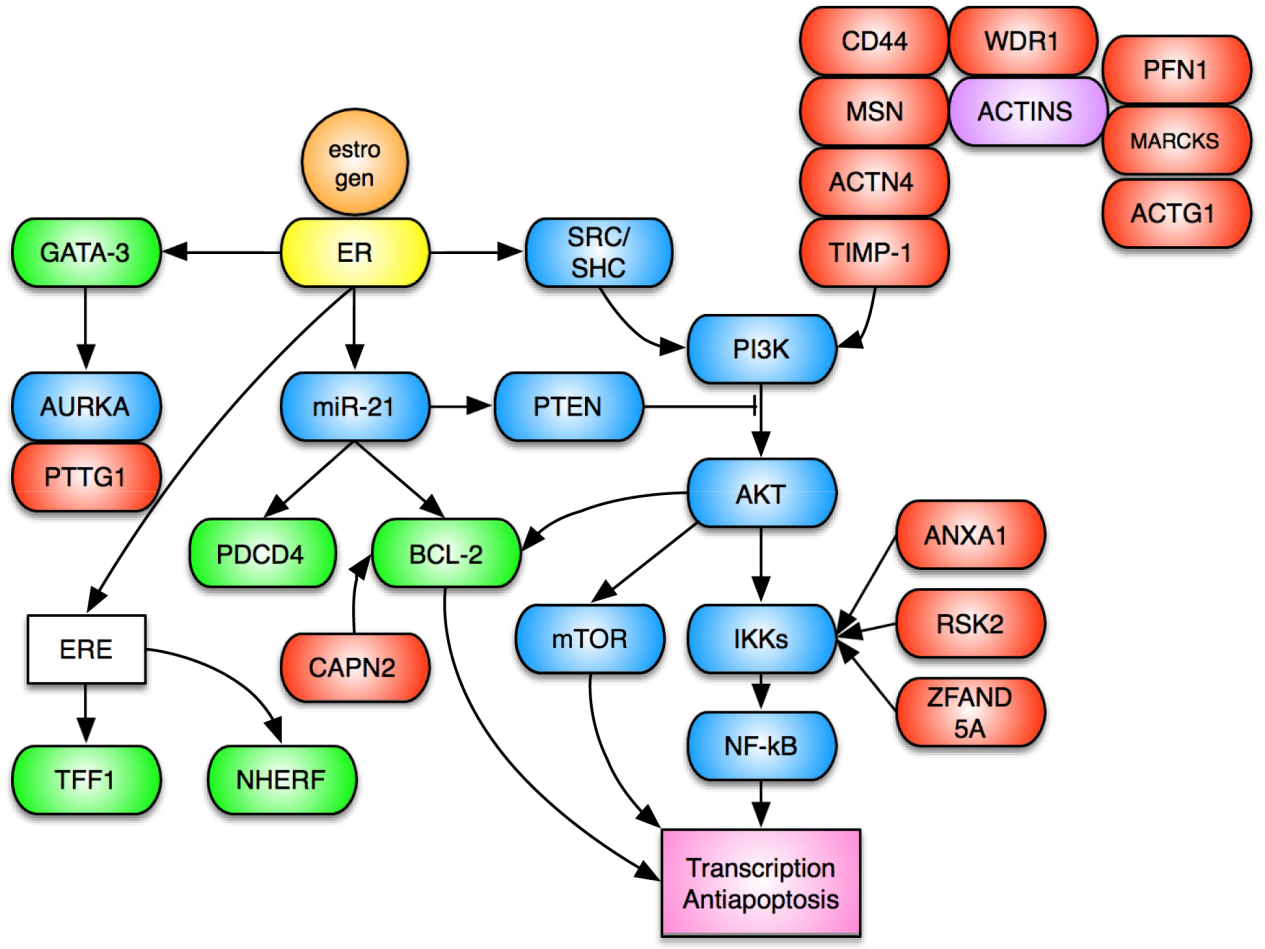
Interactions reported in the literature. Interaction between fulvestrant sensitivity genes (green) and resistance genes (red) and the known estrogen receptor signal transduction pathways (blue). Each interaction is taken from the literature and can be both at the transcription level and at the protein-protein interaction level. ERE means estrogen responsive element in the promoter of a gene. A number of resistance genes have been reported to interact with the actin cytoskeleton.

A total of 311 probesets correlated to fulvestrant resistance, meaning that they are higher expressed in cell lines resistant to fulvestrant. The top 100 probesets by correlation mapped to 78 unique genes that were subject to pathway analysis and grouped according to functional associations in Table 2. A number of these resistance genes (higher expression in resistant cell lines) modulate the PI3K signaling pathway either directly or indirectly as shown in Table 2 and also in Figure 1 [19]–[27]. They are all higher expressed in ER positive breast cancer cell lines that are resistant to fulvestrant than in ER positive cell lines that are sensitive to fulvestrant (p = 0.01 in a one-sided Wilcoxon rank test), and thus provide a potential hypothesis to explain why some ER positive cell lines are resistant to fulvestrant. Indeed, the expression of the 11-gene PI3K profile is correlated to resistance to fulvestrant in ER positive cell lines (CC = 0.76, p = 0.03). One hypothesis generated from this geneset analysis is that resistance to fulvestrant can be overcome by drugs targeting the PI3K - AKT pathway. Other resistance genes are involved in cell death more broadly defined or in response to stimulus or in cytoskeletal protein binding.

**Table 2:** Functional grouping of fulvestrant resistance genes.

Function	Genes
PI3K pathway	ANXA1, ZFAND5A, TIMP1, CD44, ACTN4, WDR1, RPS6KA3, MSN, PFN1, CD44, MARCKS
GO cell death	ANXA1, GPX1, PRNP, TIMP1, PSMB2, CD44, ACTN4, PSMD1, VIM, RPS6KA3, PFN1, YWHAB, GARS, YWHAZ, PPP2CB, MET, ACTN1, FOSL1, TNFRSF10B, FTL,PKM2, TXNRD1, F2R, CAV1, SPTAN1, TNFRSF12A
GO response to stimulus	ANXA1, GPX1, SPTBN1, ANXA2, CAPN2, PRNP, TIMP1, PSMB2, UGP2, CD44, ACTN4, MCF2L2, WDR1, PSMD1, RPS6KA3, PFN1, ASPH, YWHAB, LGALS3BP, CAV2, S100A10, PTTG1, YWHAZ, PPP2CB, MET, ACTN1, RHEB, FOSL1, TNFRSF10B, ELK3, RHOC, TXNRD1, RANBP1, F2R, CALU, STRAP, GSTO1, UPP1, ETV5, MAP4K4, TXN, PLAUR, CAV1, SPTAN1, TNFRSF12A
GO cytoskeletal protein binding	SPTBN1, ANXA2, CAPN2, TMSB10, PRNP, ACTN4, ANXA2P2, WDR1, MSN, PFN1, MARCKS, MPRIP, ACTN1, TPM4, MAPRE1, SPTAN1
Other	PSMA1, TM4SF1, SPATS2L, ETF1, ACTG1, SEPT10, RCN1, FAT1, FLJ10350, CLIC1, CARS, FKBP1A, FTL, TPI1, PKM2, FLNA, TNPO1, NUDC, LDLR, AAK1, GALNT2, EFHD2, IMP-2

### Prediction of fulvestrant sensitivity in breast cancer cell lines

**Figure 2:**
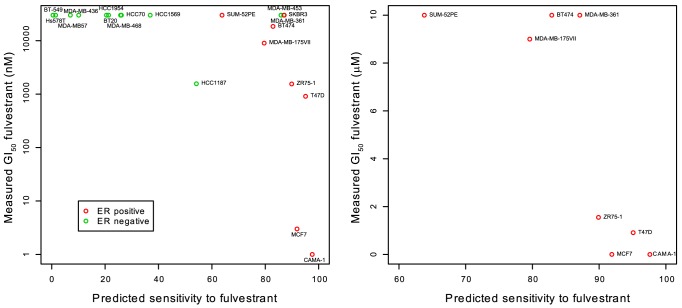
Comparison between predicted and measured sensitivity to fulvestrant for 20 breast cancer cell lines. (**A**) ERα status was determined by [28] using an immune assay or gene expression and are shown in color code (green, ERα negative; red, ERα positive). SUM-52PE is positive for ERα gene expression but negative for ERα in western blotting. The prediction score is calculated from the gene expression measurements and has been normalized to a scale of 0 to 100 (no units). If a cutoff of 50 is applied to this score, then 9 out 15 cell lines are correctly predicted as resistant (GI_50_ 5 µM or more), and 5 out of 5 cell lines are correctly predicted as sensitive. (**B**) Subgroup of ERα positive cell lines from (A) on a linear scale. GI_50_ values above 10 µM are shown as 10 µM.

To verify that the predictor developed based on the NCI60 cell line panel is indeed able to predict the sensitivity of cell lines derived from breast cancer, we blind predicted the sensitivity of 20 breast cancer cell lines based on their baseline gene expression value measured in another lab [28]. After unblinding, the prediction score was compared to the measured GI_50_ values for fulvestrant for the 20 cell lines (Figure 2). The Pearson correlation was negative 0.63, because a higher predicted sensitivity is reflected as a lower GI_50_ (P = 0.003). This was well beyond the pre-specified definition of success, a correlation of −0.30.

Figure 2a shows that all but one of the ER positive cell lines are in the 80 to 100 range for predicted senstivity, regardless of whether they are sensitive or resistant to fulvestrant. A subgroup analysis of ER positive cell lines only, however, reveals that even within ER positive cell lines, the predictor is able to differentiate cell lines based on their sensitivity to fulvestrant (CC = −0.74, P = 0.037, Figure 2b). This cannot be explained by ESR1 or PGR expression alone (CC = 0.34 and CC = −0.23, respectively, meaning that their expression is anticorrelated and weakly correlated, respectively, to fulvestrant sensitivity (GI_50_) when analyzed separately in this subgroup).

Thus, it is possible that also within ER positive breast cancer patients, the predictor will be able to distinguish between responders and non-responders to fulvestrant treatment.

### Prediction of fulvestrant sensitivity in clinical samples

**Figure 3:**
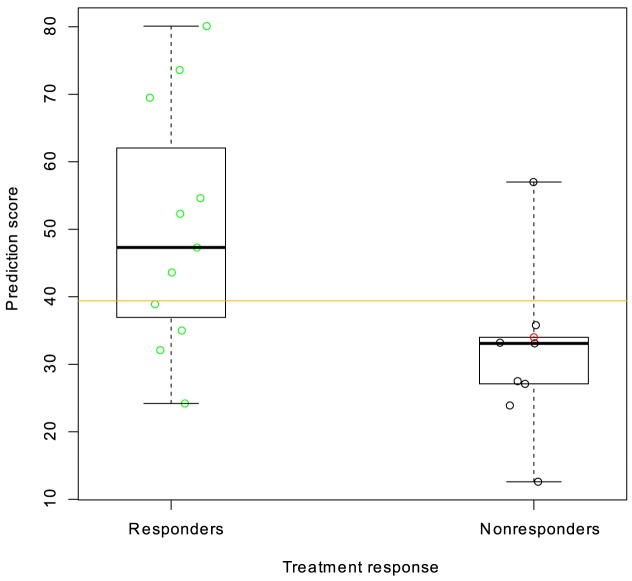
Prediction score and treatment response for the 500 mg fulvestrant cohort of the NEWEST trial. Responders are those patients that experienced a reduction in tumour size according to RECIST criteria (PR, n = 11) following treatment with 500 mg fulvestrant for 16 weeks. Non-responders were defined as those with either stable (black) or progressive disease (red) after 16 weeks treatment (SD+PD, n = 9). A one-sided Wilcoxon test for difference between predicted sensitivity of responders and non-responders yields a P-value of 0.01. The pre-specified cutoff (median of the prediction scores) is shown as an orange line. Boxes represent upper quartile, median and lower quartile.

The fulvestrant prediction score was calculated for 22 patients treated with the 500 mg-dose of fulvestrant, for whom we had pre-treatment gene expression measurements. The prediction was performed in a blinded manner and without knowledge of the clinical results. After unblinding, the predicted sensitivity of responders (PR, see Methods) was compared to the predicted sensitivity of nonresponders (SD+PD) (Figure 3). Two patients were unevaluable for response.

The correlation between predicted sensitivity and absolute reduction in Ki67 from baseline to 4 weeks was calculated as 0.32 (both 500 and 250 mg doses, one-sided p-value 0.02). This is largely a reflection of a negative correlation between the predicted sensitivity and Ki67 at 4 weeks (CC = −0.32, p = 0.02). Only tumors predicted resistant to fulvestrant had high values of Ki67 at 4 weeks. The prediction score was not significantly correlated to relative reduction in Ki67 from baseline to 4 weeks (CC = 0.15). In the clinical trial, both relative and absolute changes in Ki67 were significant after 4 weeks of treatment (47% average reduction at 250 mg dose and 79% average reduction at 500 mg dose, [4])

Standard clinicopathological features are already known for breast cancer: in particular, ER content by immunohistochemistry (H score), tumor grade, tumor size, and patient age. For this reason it was planned before unblinding that the prediction score would be compared to these covariates and the performance of the combination would be assessed as well.

When ER H score was added to absolute reduction in Ki67 the correlation of the combined score to predicted sensitivity increased to 0.41.

**Figure 4:**
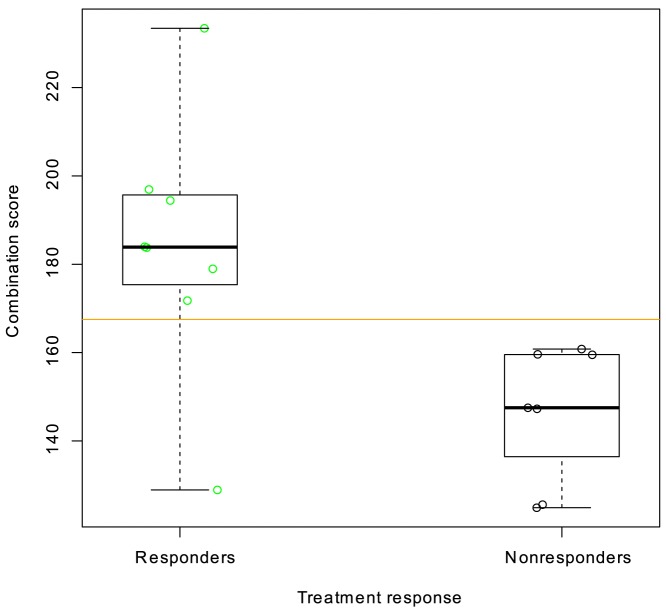
Combination score of fulvestrant sensitivity and covariates tumor grade and ER H score. Responders (PR, n = 8, green) were compared to non-responders (SD, n = 7, black). Five patients including the two with progressive disease from Figure 3 had grade information “Not done” or “unassessable” and were excluded from the analysis. A one-sided Wilcoxon test for difference between predicted sensitivity of responders and non-responders yields a P-value of 0.003. The pre-specified cutoff at the population median of the combination score is shown with an orange line. If this cutoff is used to divide this very limited sample of patients into predicted sensitive and predicted resistant to fulvestrant, the PPV of the prediction is 88% and the NPV of this prediction is 100%.

The predicted sensitivity was combined with other covariates as described in the methods section. Figure 4 shows the score obtained by combining the prediction score with tumor grade and ER H score for the 500 mg dose patients (the combination score). It is evident that this combination score gives a better separation between responders and non-responders than that obtained by comparing the individual values with response. Although all patients were selected for inclusion based on a categorization as ER positive, a more quantitative determination of ER receptor status in the H score obviously contains information that differentiates between fulvestrant responsive and unresponsive patients. The same holds true for tumor grade.

**Figure 5:**
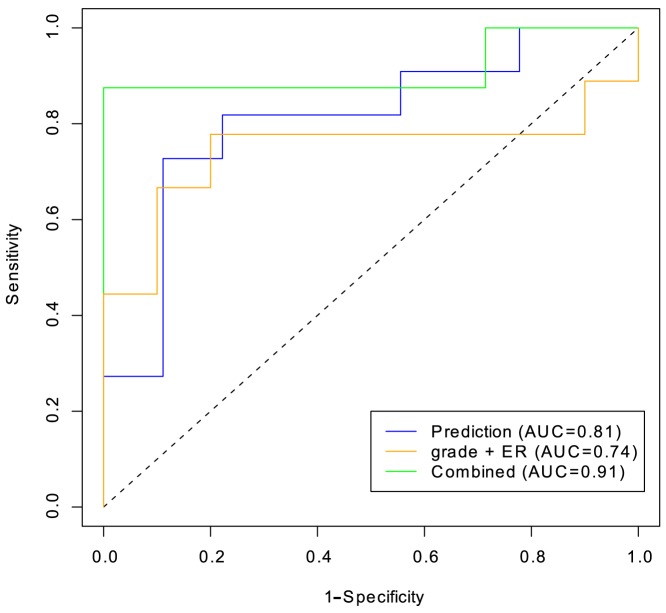
A receiver operating characteristic comparing three prediction scores: grade +ER (orange), prediction score (blue) and combination score (green). The dashed line shows an AUC of 0.5.

The different predictors were compared by measuring the Area Under the Curve (AUC) in a Receiver- Operating Characteristic (ROC). Figure 5 shows how the tradeoff between sensitivity and specificity varies with all possible cutoffs used. It can be seen that the prediction score appears to be a more accurate predictor (AUC 0.81, 95% CI 0.6–1.0) than the combination of clinical covariates ER H score and tumor grade (AUC 0.74, 95% CI 0.47–1.0), but the combination of prediction score and covariates is superior (AUC 0.91, 95% CI 0.73–1.0). The difference in AUC between combination score and grade +ER score was not statistically significant in this limited sample size.

**Figure 6:**
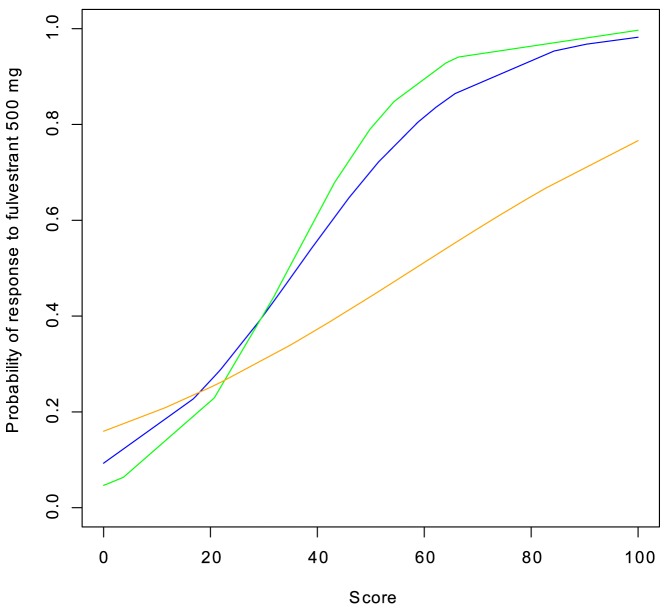
Logistic regression of the relationship between fulvestrant prediction score and probability of response. Three predictors are shown: grade +ER (orange), prediction score (blue) and combination score (green). The 95% confidence intervals are quite high (not shown) due to the limited sample size but a Wald test on the logistic regression of the prediction score is borderline statistically significant (P = 0.0499), as is the combination score (P = 0.0475). All scores have been normalized to a scale from 0 to 100 for comparison.

Dividing patients into predicted sensitive and predicted resistant after applying a cutoff loses valuable information from the quantitative prediction score. A patient with a prediction score of 100 has a higher probability of responding to treatment than a patient with a prediction score of 60 even though they are both classified as sensitive to fulvestrant. This information can be visualized in a logistic regression which converts the prediction score into a probability of response to treatment. Figure 6 shows such logistic regression curves for the three prediction scores: grade +ER, prediction score and combination score. Again, the combination score is superior by giving a higher range of probabilities (from 5% to 99.66%) of response to fulvestrant treatment. It is only marginally better than the prediction score, however (ranging from 9% to 98% probability of response).

### Comparison to other gene expression signatures for breast cancer

A number of prognostic and predictive signatures have been developed specifically for breast cancer. Among these are the PAM50 gene list that divides patients into the intrinsic subtypes of luminal A, luminal B, Her2, basal and normal. Applying the PAM50 matrix from [12] to the baseline samples from the 500 mg group indicated that there were 7 luminal A-like, 12 luminal B-like, and one normal-like (this is a consequence of the PAM50 training set containing normal breast samples). The luminal subtypes did not correlate significantly with clinical response (fisher P = 0.07), nor did they contribute in a multivariate regression model including tumor grade and ER score. When subtypes were used to create a Risk-of-Relapse (ROR) score as described by Parker [12], there was no correlation of the ROR score with post-treatment change in Ki67 (P = 0.94).

Parker [12] observed that patients with a higher ROR score had a higher probability of clinical response to neoadjuvant treament with chemotherapy T/FAC. Similarly, the ROR score is associated with a higher probability of response to treatment with fulvestrant in our cohort (AUC 0.74, 95% CI 0.54–0.94). Combining the ROR score with clinical covariates grade and ER H score did not improve the association within the NEWEST dataset(AUC 0.73, 95% CI: 0.48–0.99). This suggests that the predictive information in the ROR score is already present in the clinical covariates (AUC 0.74, 95% CI 0.47–1.0).

The Oncotype DX signature is used to predict the risk of recurrence in early breast cancer. The recurrence score was significantly correlated to absolute Ki67 measurements before (P = 3×10^−5^) and 4 weeks after treatment (P = 0.03), but was not correlated to absolute (P = 0.86) or relative (P = 1.0) change in Ki67 during treatment. The recurrence score did not contribute to prediction of response to treatment in a multivariate model.

A published signature [29] of zinc-finger transcription factor induced fulvestrant resistance had minimal overlap to our signature. Only 5 out of 95 probesets in the published fulvestrant resistance signature were among the probesets used in our fulvestrant prediction score, and the published 95 probesets could not predict response in our fulvestrant treated cohort (P = 0.29 in a one-sided Wilcoxon test between responder scores and nonresponder scores).

### Analysis of 250 mg fulvestrant regimen

**Figure 7:**
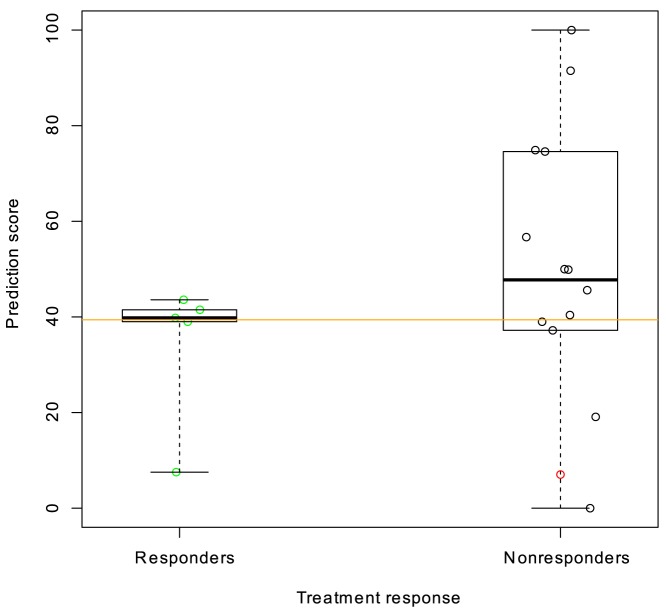
The prediction score for patients treated with 250 mg fulvestrant. There are 5 partial responders (green), 13 patients with stable disease (black) and 1 progressive disease patient (red). The median prediction score, as shown in Figure 2, is represented by the yellow line in the graph. Most patients predicted to be sensitive (above cutoff indicated by yellow line, population median as shown in Figure 2) exhibited stable disease at this dose, whereas they exhibited partial response at the 500 mg dose (Figure 3).

Array data and outcome information was available for 19 patients who received the lower dose of 250 mg fulvestrant in the NEWEST trial. Figure 7 shows the predicted sensitivity score for responders and non-responders treated with 250 mg fulvestrant. Among these 19 patients, the overall response rate (CR+PR) was only half of that observed in those treated with the 500 mg dose, 26% vs. 50%. There is a wide range of prediction scores in the nonresponding group, and the average score of this group is not significantly different from the responders. Although the reasons behind the lack of difference in prediction score between the two groups is not clear, it is possible that it is due to the small group size and the fact that a lower dose of fulvestrant was given, which produced a smaller drop in tumour Ki67 levels in this trial [4].

### Prediction of response to other endocrine agents

We tested whether the fulvestrant response prediction score could also predict response to aromatase inhibition in a small cohort of 21 patients treated with neoadjuvant anastrozole in a similar protocol to that used to test fulvestrant [5]. There was no correlation between predicted sensitivity to fulvestrant and the overall response to anastrozole. Nine responders had an average predicted sensitivity of 52 (95% CI 20–84). Seven nonresponders had an average predicted sensitivity of 51 (95% CI 4–97) In addition there was no correlation between prediction score and change in Ki67 produced after 2 weeks of anastrozole treatment (N = 21, P = 0.9).

**Figure 8:**
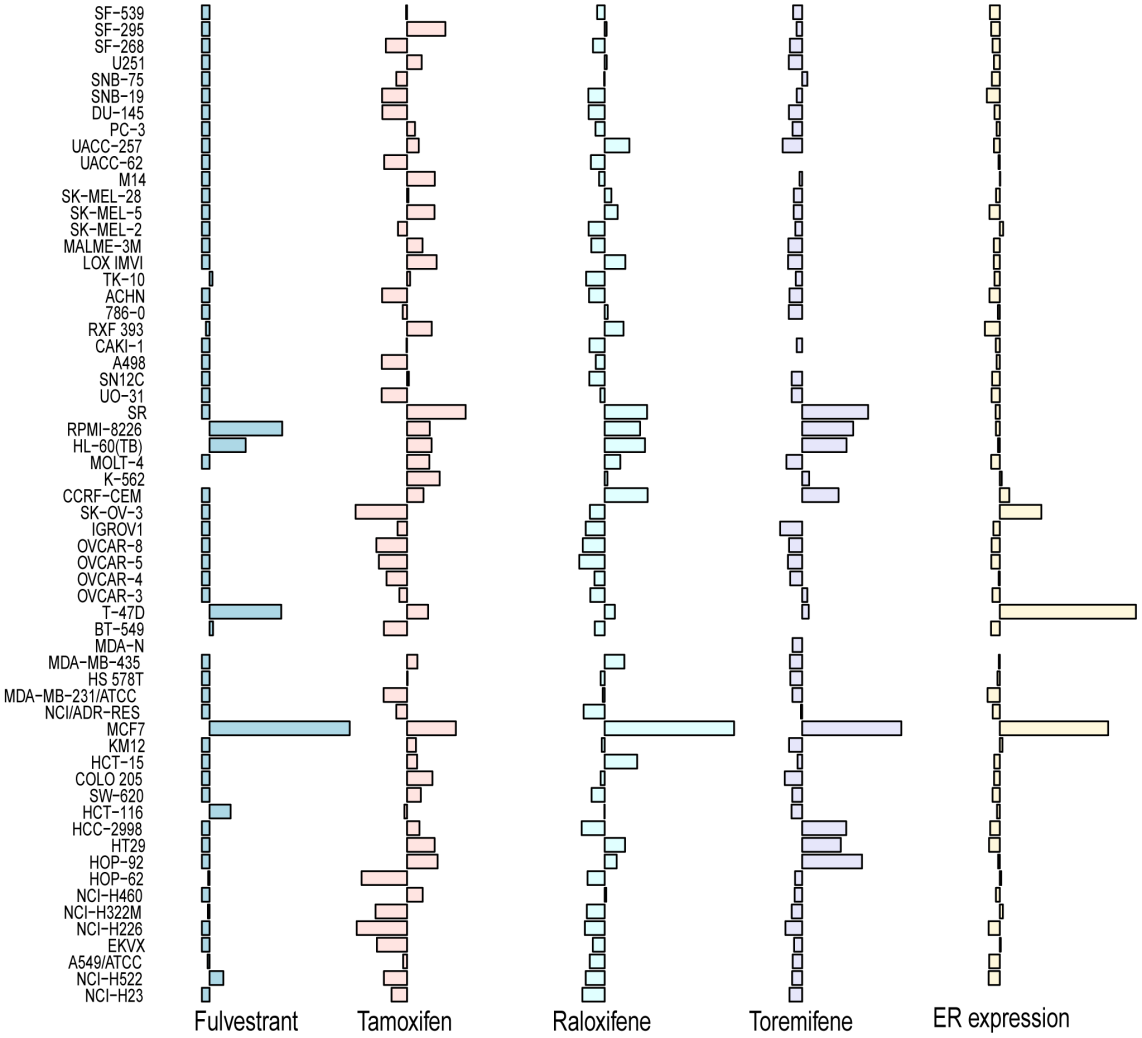
Differences in the sensitivity (GI50) of NCI60 cell lines to drugs targeting the ER pathway. For each drug, and each cell line, the GI50 is shown as a bar on a logarithmic scale relative to the mean GI50 of all cell lines (63 µM for fulvestrant, 4.3 µM for tamoxifen, 8.4 µM for raloxifene, 13 µM for toremifene). Bars to the right of the mean indicate above average sensitivity cell lines, whereas bars to the left of the mean indicate below average sensitivity cell lines. ER expression is measured with an Affymetrix array on all cell lines and shown on a logarithmic scale relative to the mean expression. MCF7 and T-47D are well known ER positive cell lines.

Figure 8 shows how fulvestrant, tamoxifen and other agents targeting ER differ in their in vitro effects on the same NCI60 cell line panel. For fulvestrant, cell lines with a GI_50_ of less than 5 µM are considered sensitive. Three cell lines are sensitive by this definition, and two of them have a high expression of the ER gene (*ESR1*). However, the data suggests that that other factors can contribute to fulvestrant sensitivity in vitro. These may be revealed by a study of the resistance and sensitivity determining genes found in this study. It is evident from Figure 8 that fulvestrant differs in sensitivity profile from tamoxifen, raloxifene and toremifene. Indeed the fulvestrant prediction score is better than the other *in vitro* based prediction scores at predicting the 500 mg dose fulvestrant clinical data of Figure 3: fulvestrant score: P = 0.010, tamoxifen score: P = 0.012, anastrozole score: P = 0.56, ER expression (ESR1 gene): P = 0.10. The fact that the tamoxifen score can predict response to fulvestrant is not surprising as, despite differences in their in vitro profile (Figure 8), the two drugs target the same molecule.

## Discussion

We describe here a gene expression prediction score derived from an analysis of cultured cell lines treated with fulvestrant that when applied to clinical tissues may enrich for response in breast cancer patients treated with neoadjuvant fulvestrant. This raises the prospect of developing a prospective score that may identify those patients most likely to respond to fulvestrant treatment and those most likely to be resistant. There are potential clinical benefits for both groups: fulvestrant sensitive patients as defined by the gene expression score may have a much greater probability of responding in terms of reduction in tumour size and prolonged PFS. Patients predicted to be fulvestrant-insensitive may be spared treatment that is unlikely to be effective and can be redirected to other, potentially more appropriate regimens.

The concentration of fulvestrant used in vitro (10^−8^ to 10^−4^ M in the NCI60 panel, 10^−9^ to 10^−5^ M in the breast cancer cell line panel) is slightly higher than that measured in plasma from patients (10^−9^ to 10^−8^ M [30]). It is not uncommon that higher concentrations are needed to achieve a measurable effect *in vitro* during the 48 hours of the *in vitro* assay.

The resistance genes shown in Figure 1 could be used to suggest methods to overcome fulvestrant resistance. As the resistance genes primarily act through the PI3K signal transduction pathway, agents that target PI3K should inhibit cells resistant to fulvestrant. This suggests that combining fulvestrant with a PI3K inhibitor drug could increase the response rate further. This has been suggested before [31] and demonstrated *in vitro* [32], where PI3K inhibitor LY294002 was able to enhance the cytostatic effect of fulvestrant, and in [33] where combined treatment with AKT-inhibitor AZD5363 and fulvestrant suppressed MCF-7 xenograft growth better than either drug alone. Combination of PI3K inhibitor and fulvestrant is currently being tested clinically [34]. The addition of the mTOR inhibitor everolimus to endocrine therapy has been shown in three clinical trials to improve response rate or survival in ER-positive breast cancer [35]–[37].

One cell line was sensitive to fulvestrant despite low expression of ESR1, and two patients responded to treatment with fulvestrant despite a low ER H score (0–1 on scale of 0 to 300). This suggests that fulvestrant may trigger apoptosis in ER negative cells, and this has been demonstrated in vitro on ER negative cells [38]. The mechanisms of fulvestrant-induced apoptosis may be searched among the Table 1 genes that respond to stimulus. Of these, BCL-2 and TNFRs have previously been implicated in apoptosis induced by fulvestrant [39].

The biological relevance filter applied to the predictor genes has the effect of removing false positive correlated genes but includes a risk of missing important pathways (false negative genes). In the absence of the biological relevance filter the prediction score is no longer significantly correlated to patient response (p = 0.06), why we chose to trust only those genes that passed the biological relevance filter.

Mutations in ESR1 have recently been found in ER-positive breast cancer patients and can explain endocrine resistance [40]–[41]. We have not sequenced the ESR1 gene in our patients and cell lines.

The clinical sample size used in this study to test a score derived on preclinical data is small. It will therefore be important to determine whether this gene expression score for fulvestrant response will retain its predictive power when tested on tissues from a larger cohort of patients treated with fulvestrant, particularly when applied to tumors from patients with advanced and recurrent breast cancer. In the advanced and recurrent setting, it is critical to compare the prediction score to progression free survival. It remains critical to determine the level of discordance between prediction scores obtained from individual diagnostic primary tumor samples and biopsies from recurrent metastatic disease. Finally, the ability to detect and quantify this predictive score should be tested and validated using formalin-fixed paraffin embedded (FFPE) samples instead of fresh tumor samples, in order to maximize the potential clinical utility of this predictive score in the future.

## References

[1] Di Leo A, Jerusalem G, Petruzelka L, Torres R, Bondarenko IN, et al (2010) Results of the CONFIRM phase III trial comparing fulvestrant 250 mg with fulvestrant 500 mg in postmenopausal women with estrogen receptor-positive advanced breast cancer. J Clin Oncol. 28(30): 4594–600. 20855825 10.1200/JCO.2010.28.8415

[2] Robertson JF, Llombart-Cussac A, Rolski J, Feltl D, Dewar J, et al (2009) Activity of fulvestrant 500 mg versus anastrozole 1 mg as first-line treatment for advanced breast cancer: results from the FIRST study. J Clin Oncol. 27(27): 4530–5. 19704066 10.1200/JCO.2008.21.1136

[3] Paik S, Shak S, Tang G, Kim C, Bakker J, et al (2004) A Multigene Assay to Predict Recurrence of Tamoxifen-Treated, Node-Negative Breast Cancer”. N Engl J Med 351(27): 2817–2826. 15591335 10.1056/NEJMoa041588

[4] Kuter I, Gee JM, Hegg R, Singer CF (2012) Dose-dependent change in biomarkers during neoadjuvant endocrine therapy with fulvestrant: results from NEWEST, a randomized Phase II study. Breast Cancer Res Treat 133(1): 237–246. 10.1007/s10549-011-1947-7 22286314

[5] Smith IE, Walsh G, Skene A, Llombart A, Mayordomo JI, et al (2007) A phase II placebo-controlled trial of neoadjuvant anastrozole alone or with gefitinib in early breast cancer. J Clin Oncol. 25(25): 3816–22. 17679728 10.1200/JCO.2006.09.6578

[6] Chen JJ, Knudsen S, Mazin W, Dahlgaard J, Zhang B (2012) A 71-gene signature of TRAIL sensitivity in cancer cells. Mol Cancer Ther. 11(1): 34–44. 22027696 10.1158/1535-7163.MCT-11-0620

[7] Wang W, Baggerly KA, Knudsen S, Askaa J, Mazin W, et al (2013) Independent validation of a model using cell line chemosensitivity to predict patient response to cancer therapeutics. J. Natl. Cancer Inst. 105(17): 1284–91. 23964133 10.1093/jnci/djt202 PMC3955959

[8] Shankavaram UT, Reinhold WC, Nishizuka S, Major S, Morita D, et al (2007) Transcript and protein expression profiles of the NCI-60 cancer cell panel: an integromic microarray study. Mol Cancer Ther 6: 820–32. 17339364 10.1158/1535-7163.MCT-06-0650

[9] Chuang HY, Lee E, Liu YT, Lee D, Ideker T (2007) Network-based classification of breast cancer metastasis. Mol Syst Biol. 3: 140. 17940530 10.1038/msb4100180 PMC2063581

[10] Reimand J, Kull M, Peterson H, Hansen J, Vilo J (2007) g:Profiler–a web-based toolset for functional profiling of gene lists from large-scale experiments. Nucleic Acids Res. 35(Web Server issue):W193–200. 10.1093/nar/gkm226 PMC1933153 17478515

[11] Irizarry RA, Hobbs B, Collin F, Beazer-Barclay YD, Antonellis KJ, et al (2003) Exploration, normalization, and summaries of high density oligonucleotide array probe level data. Biostatistics 4(2): 249–64. 12925520 10.1093/biostatistics/4.2.249

[12] Parker JS, Mullins M, Cheang MC, Leung S, Voduc D, et al (2009) Supervised risk predictor of breast cancer based on intrinsic subtypes. J Clin Oncol. 27(8): 1160–7. 19204204 10.1200/JCO.2008.18.1370 PMC2667820

[13] Robin X, Turck N, Hainard A, Tiberti N, Lisacek F, et al. (2011) pROC: an open-source package for R and S+ to analyze and compare ROC curves. BMC Bioinformatics, 12 , p. 77. 10.1186/1471-2105-12-77 PMC3068975 21414208

[14] Jiang S, Katayama H, Wang J, Li SA, Hong Y, et al (2010) Estrogen-induced aurora kinase-A (AURKA) gene expression is activated by GATA-3 in estrogen receptor-positive breast cancer cells. Horm Cancer. 1(1): 11–20. 21761347 10.1007/s12672-010-0006-x PMC4501777

[15] Li Y, Sun L, Zhang Y, Wang D, Wang F, et al (2011) The histone modifications governing TFF1 transcription mediated by estrogen receptor. J Biol Chem. 286(16): 13925–36. 21378170 10.1074/jbc.M111.223198 PMC3077593

[16] Perillo B, Sasso A, Abbondanza C, Palumbo G (2000) 17beta-estradiol inhibits apoptosis in MCF-7 cells, inducing bcl-2 expression via two estrogen-responsive elements present in the coding sequence. Mol Cell Biol. 20(8): 2890–901. 10733592 10.1128/mcb.20.8.2890-2901.2000 PMC85519

[17] Stemmer-Rachamimov AO, Wiederhold T, Nielsen GP, James M, Pinney-Michalowski D, et al (2001) NHE-RF, a merlin-interacting protein, is primarily expressed in luminal epithelia, proliferative endometrium, and estrogen receptor-positive breast carcinomas. Am J Pathol. 158(1): 57–62. 11141479 10.1016/S0002-9440(10)63944-2 PMC1850244

[18] Wickramasinghe NS, Manavalan TT, Dougherty SM, Riggs KA, Li Y, et al (2009) Estradiol downregulates miR-21 expression and increases miR-21 target gene expression in MCF-7 breast cancer cells. Nucleic Acids Res. 37(8): 2584–95. 19264808 10.1093/nar/gkp117 PMC2677875

[19] Bist P, Leow SC, Phua QH, Shu S, Zhuang Q, et al (2011) Annexin-1 interacts with NEMO and RIP1 to constitutively activate IKK complex and NF-κB: implication in breast cancer metastasis. Oncogene 30(28): 3174–85. 21383699 10.1038/onc.2011.28

[20] Huang J, Teng L, Li L, Liu T, Li L, et al (2004) ZNF216 Is an A20-like and IkappaB kinase gamma-interacting inhibitor of NFkappaB activation. J Biol Chem. 279(16): 16847–53. 14754897 10.1074/jbc.M309491200

[21] Fu ZY, Lv JH, Ma CY, Yang DP, Wang T (2011) Tissue inhibitor of metalloproteinase-1 decreased chemosensitivity of MDA-435 breast cancer cells to chemotherapeutic drugs through the PI3K/AKT/NF-кB pathway. Biomed Pharmacother. 65(3): 163–7. 10.1016/j.biopha.2011.02.004 21684102

[22] Ju JH, Jang K, Lee KM, Kim M, Kim J, et al (2011) CD24 enhances DNA damage-induced apoptosis by modulating NF-κB signaling in CD44-expressing breast cancer cells. Carcinogenesis 32(10): 1474–83. 21798852 10.1093/carcin/bgr173

[23] Sunohara JR, Ridgway ND, Cook HW, Byers DM (2001) Regulation of MARCKS and MARCKS-related protein expression in BV-2 microglial cells in response to lipopolysaccharide. J Neurochem. 78(3): 664–72. 11483670 10.1046/j.1471-4159.2001.00458.x

[24] Wang J, Yuan Y, Zhou Y, Guo L, Zhang L, et al (2008) Protein interaction data set highlighted with human Ras-MAPK/PI3K signaling pathways. J Proteome Res. 7(9): 3879–89. 18624398 10.1021/pr8001645

[25] Peng C, Cho YY, Zhu F, Xu YM, Wen W, et al (2010) RSK2 mediates NF-kappaB activity through the phosphorylation of IkappaBalpha in the TNF-R1 pathway. FASEB J. 24(9): 3490–9. 20385620 10.1096/fj.09-151290 PMC2923348

[26] Caglayan E, Romeo GR, Kappert K, Odenthal M, Südkamp M, et al (2010) Profilin-1 is expressed in human atherosclerotic plaques and induces atherogenic effects on vascular smooth muscle cells. PLoS One 5(10): e13608. 21049052 10.1371/journal.pone.0013608 PMC2963617

[27] Babakov VN, Petukhova OA, Turoverova LV, Kropacheva IV, Tentler DG, et al (2008) RelA/NF-kappaB transcription factor associates with alpha-actinin-4. Exp Cell Res. 314(5): 1030–8. 18215660 10.1016/j.yexcr.2007.12.001

[28] Neve RM, Chin K, Fridlyand J, Yeh J, Baehner FL, et al (2006) A collection of breast cancer cell lines for the study of functionally distinct cancer subtypes. Cancer Cell. 10(6): 515–27. 17157791 10.1016/j.ccr.2006.10.008 PMC2730521

[29] Lee J, Hirsh AS, Wittner BS, Maeder ML, Singavarapu R, et al (2011) Induction of stable drug resistance in human breast cancer cells using a combinatorial zinc finger transcription factor library. PLoS One. 6(7): e21112. 21818254 10.1371/journal.pone.0021112 PMC3139592

[30] Robertson JF, Erikstein B, Osborne KC, Pippen J, Come SE, et al (2004) Pharmacokinetic profile of intramuscular fulvestrant in advanced breast cancer. Clin Pharmacokinet. 43(8): 529–38. 15170367 10.2165/00003088-200443080-00003

[31] Fox EM, Arteaga CL, Miller TW (2012) Abrogating endocrine resistance by targeting ERα and PI3K in breast cancer. Front Oncol. 2: 145. 23087906 10.3389/fonc.2012.00145 PMC3472546

[32] Ghayad SE, Vendrell JA, Ben Larbi S, Dumontet C, Bieche I, et al (2010) Endocrine resistance associated with activated ErbB system in breast cancer cells is reversed by inhibiting MAPK or PI3K/Akt signaling pathways. Int J Cancer. 126(2): 545–62. 19609946 10.1002/ijc.24750

[33] Fox EM, Kuba MG, Miller TW, Davies BR, Arteaga CL (2013) Autocrine IGF-I/insulin receptor axis compensates for inhibition of AKT in ER-positive breast cancer cells with resistance to estrogen deprivation. Breast Cancer Res. 15(4): R55. 23844554 10.1186/bcr3449 PMC3979036

[34] Di Leo A, Germa C, Weber D, Di Tomaso E, Dharan B, et al.. (2012) Phase III randomized study of the oral pan-PI3K inhibitor BKM120 with fulvestrant in postmenopausal women with HR+/HER2– locally advanced or metastatic breast cancer, treated with aromatase inhibitor, and progressed on or after mTOR inhibitor-based treatment – BELLE-3. Cancer Res 72(24 Suppl):Abstract nr OT2-3-08.

[35] Baselga J, Semiglazov V, van Dam P, Manikhas A, Bellet M, et al (2009) Phase II randomized study of neoadjuvant everolimus plus letrozole compared with placebo plus letrozole in patients with estrogen receptor-positive breast cancer. J Clin Oncol. 27(16): 2630–7. 19380449 10.1200/JCO.2008.18.8391

[36] Bachelot T, Bourgier C, Cropet C, Guastalla J.-P, Ferrero J-M, et al. (2010) TAMRAD: a GINECO randomized phase II trial of everolimus in combination with tamoxifen versus tamoxifen alone in patients (pts) with hormone-receptor positive, HER2 negative metastatic breast cancer (MBC) with prior exposure to aromatase inhibitors (AI). Cancer Res. 70 abstract S1–S6. 10.1200/JCO.2011.39.0708 22565002

[37] Baselga J, Campone M, Piccart M, Burris HA III, Rugo HS, et al (2012) Everolimus in postmenopausal hormone-receptor-positive advanced breast cancer. N Engl J Med. 366(6): 520–9. 22149876 10.1056/NEJMoa1109653 PMC5705195

[38] Treon SP, Teoh G, Urashima M, Ogata A, Chauhan D, et al (1998) Anti-estrogens induce apoptosis of multiple myeloma cells. Blood. 92(5): 1749–57. 9716605

[39] Smolnikar K, Löffek S, Schulz T, Michna H, Diel P (2000) Treatment with the pure antiestrogen faslodex (ICI 182780) induces tumor necrosis factor receptor 1 (TNFR1) expression in MCF-7 breast cancer cells. Breast Cancer Res Treat. 63(3): 249–59. 11110059 10.1023/a:1006490416408

[40] Toy W, Shen Y, Won H, Green B, Sakr RA, et al.. (2013) ESR1 ligand-binding domain mutations in hormone-resistant breast cancer. Nat Genet. 2013 Nov 3. doi: 10.1038/ng.2822. 10.1038/ng.2822 PMC3903423 24185512

[41] Robinson DR, Wu YM, Vats P, Su F, Lonigro RJ, et al.. (2013) Activating ESR1 mutations in hormone-resistant metastatic breast cancer. Nat Genet. 2013 Nov 3. doi: 10.1038/ng.2823. 10.1038/ng.2823 PMC4009946 24185510

